# Development and validation of immunotherapy nomogram for predicting the efficacy and prognosis of recurrent and metastatic cervical cancer

**DOI:** 10.3389/fimmu.2025.1662605

**Published:** 2025-12-08

**Authors:** Zhongan Liu, Sijia Zhang, Yan Wang, Yan Zong, Tian Zhou, Daying Wu, Ganxin Wang, Lihong He, Kai Huang, Yunqing Xu, Quan Tang, Mulan Chen, Guangqin Xiao, Guiling Li

**Affiliations:** 1 Cancer Center, Union Hospital, Tongji Medical College, Huazhong University of Science and Technology, Wuhan, China; 2 Institute of Radiation Oncology, Union Hospital, Tongji Medical College, Huazhong University of Science and Technology, Wuhan, China; 3 Hubei Key Laboratory of Precision Radiation Oncology, Wuhan, China; 4 Chongqing Hospital, Union Hospital, Tongji Medical College, Huazhong University of Science and Technology, Chongqing, China; 5 Department of Biophysics, Center for Integrative Physiology and Molecular Medicine (CIPMM), School of Medicine, Saarland University, Homburg, Germany; 6 Department of Biomedical Sciences, Institute for Health Research and Education, Osnabrück University, Osnabrück, Germany; 7 Department of Oncology, Liyuan Hospital, Tongji Medical School, Huazhong University of Science and Technology, Wuhan, China; 8 Department of Infectious Diseases, Union Hospital, Tongji Medical College, Huazhong University of Science and Technology, Wuhan, China; 9 Department of Oncology, People’s Hospital of Huangpi District, Jianghan University, Wuhan, China; 10 Department of Oncology, Hubei Aerospace Hospital, Xiaogan, China; 11 Department of Epidemiology, Harvard T.H. Chan School of Public Health, Boston, MA, United States; 12 Clinical and Translational Epidemiology Unit, Massachusetts General Hospital and Harvard Medical School, Boston, MA, United States

**Keywords:** cervical cancer, immunotherapy, nomogram, biomarker, PD-L1

## Abstract

**Background:**

Cervical cancer, a major cause of cancer-related mortality in women, remains challenging to treat, particularly in recurrent or metastatic stages. Immunotherapy offers promise, but reliable biomarkers for predicting outcomes are lacking.

**Method:**

A cohort of 204 patients with recurrent or metastatic cervical cancer who underwent immunotherapy was included in the study. Predictive factors were identified using LASSO regression combined with multivariate Cox proportional hazards analysis. Nomograms for progression-free survival (PFS) and overall survival (OS) were constructed by incorporating significant prognostic variables. Internal validation was performed via bootstrap resampling, and clinical applicability was assessed through decision curve analysis (DCA). Risk stratification was evaluated using Kaplan-Meier survival curves, with log-rank tests for comparison. In addition, to enhance the clinical applicability of the model, we downloaded external multi-center validation datasets from public databases, including TCGA (The Cancer Genome Atlas) and GEO (Gene Expression Omnibus). Data from 306 cervical cancer patients were obtained and used as an independent validation cohort. These external datasets allowed for the verification of the developed model, ensuring its robustness and generalizability across different clinical settings.

**Results:**

The analysis identified several significant prognostic factors for PFS, including histological type, maximum lesion diameter, CA125 levels, albumin concentration, lactate dehydrogenase (LDH) activity, and neutrophil-to-lymphocyte ratio (NLR). For OS, independent factors included BMI, liver metastasis, CEA levels, hemoglobin concentration, albumin levels, and LDH. The nomogram models demonstrated strong predictive accuracy, with concordance indices (C-index) of 0.706 for PFS and 0.769 for OS. Calibration curves indicated that the predicted and actual outcomes were in excellent agreement. The area under the curve (AUC) values for PFS at 1- and 2-year follow-up were 0.804 and 0.822, respectively, while for OS, the AUC values were 0.880 and 0.781. The risk stratification based on the nomogram scores revealed significant survival differences between high- and low-risk patients, with the high-risk group exhibiting poorer survival outcomes. External validation using data from the TCGA and GEO cohorts confirmed the robustness and generalizability of the nomogram models, further supporting their clinical relevance.

**Conclusion:**

These nomograms provide a reliable tool for predicting outcomes in cervical cancer immunotherapy, helping to personalize treatment and improve clinical management, especially for metastatic disease.

## Introduction

Cervical cancer is the most prevalent gynecological malignancy worldwide and remains one of the leading causes of cancer-related mortality in women (1). While the advent of surgery, concurrent chemoradiotherapy, and intrauterine irradiation has significantly improved the overall response rates and survival outcomes for cervical cancer patients, these treatments have limited efficacy in cases of recurrent or metastatic disease (2). The median survival for patients with advanced cervical cancer is approximately 16.8 months, with an overall five-year survival rate of less than 20% (3). In recent years, immune checkpoint inhibitors have garnered increasing attention and are being integrated into the treatment regimens for various solid tumors, offering a promising and well-tolerated therapeutic option.

Cervical cancer is a virus-induced neoplasm, driven primarily by the persistent infection of high-risk human papillomavirus (HPV), leading to the overexpression of oncoproteins E6 and E7 in tumor epithelial cells. This upregulation facilitates immune evasion, notably through the overexpression of programmed death ligand 1 (PD-L1), which contributes to immune escape (4). Given these immunogenic characteristics, cervical cancer has been recognized as an immunodominant malignancy, with a higher potential for response to immunotherapy. However, despite these promising features, clinical response rates to immunotherapy in cervical cancer remain modest, ranging from 10% to 25%, with a significant portion of patients experiencing treatment failure due to tumor recurrence and metastasis, ultimately resulting in poor long-term survival outcomes (5). Thus, there is a critical need to identify reliable and effective biomarkers to predict both the therapeutic response and prognosis of immunotherapy in cervical cancer patients, as well as to identify subgroups that may benefit most from this treatment.

Several studies have highlighted the influence of clinicopathological factors on the efficacy and prognosis of immunotherapy in cancer patients, including histologic subtype, tumor size, lymph node metastasis, and biomarkers such as lactate dehydrogenase (LDH) levels (6–8). Various predictive models have been developed to identify patient populations that may derive benefit from immunotherapy. For example, a model incorporating the pre-treatment performance status (PS) score, alkaline phosphatase, and hemoglobin levels demonstrated superior prognostic discrimination for immunotherapy response in patients with advanced urothelial carcinoma (9). Another immune-prognostic scoring system, utilizing albumin, LDH, and the neutrophil-to-lymphocyte ratio (NLR), has shown good clinical applicability in selecting appropriate candidates for phase I immunotherapy trials (10). However, the prognostic value of these clinicopathological factors in predicting the response to immunotherapy in advanced cervical cancer remains unclear. Moreover, despite the availability of various predictive models in other cancer types, few studies in the field of cervical cancer immunotherapy have employed nomogram-based visual prediction tools that integrate multiple clinicopathological predictors.

In this study, we aim to explore the prognostic significance of these predictive factors in the context of immunotherapy for advanced cervical cancer. Our goal is to develop a novel predictive nomogram to assist clinicians in assessing the individualized prognosis and therapeutic outcomes of immunotherapy for cervical cancer patients.

## Materials and methods

### Patients

This retrospective study included 300 patients who received immunotherapy for recurrent and metastatic cervical cancer at the Cancer Center, Union Hospital, Tongji Medical College, Huazhong University of Science and Technology between March 2018 and August 2024. The inclusion criteria were patients diagnosed with recurrent or metastatic cervical cancer who underwent immunotherapy during the study period. The exclusion criteria were as follows (1): patients with cervical cancer and a second primary malignancy, and (2) patients who received fewer than two cycles of treatment or had incomplete follow-up data. After applying these exclusions, a total of 204 patients with complete clinical and pathological data were included in the final analysis. The median follow-up duration for these patients was 39 months, with the shortest follow-up being 4 months and the longest 135 months. This study was conducted in accordance with the ethical principles outlined in the Declaration of Helsinki. Ethical approval was granted by the Institutional Review Board and Ethics Committee of Cancer Center, Union Hospital, Tongji Medical College, Huazhong University of Science and Technology *(Approval number: UHCT230829)*.

### Clinicopathology data collection

In this study, a total of 20 clinicopathological variables were included to minimize potential confounders. These factors comprised demographic and clinical characteristics, as well as laboratory and inflammatory biomarkers. Specific cut-off values were used to define the elevated levels of key markers: CA125 was considered elevated if ≥35 U/mL, CEA ≥5 ng/mL, LDH ≥250 U/L, hemoglobin <12 g/dL (for anemia), albumin <3.5 g/dL (for hypoalbuminemia), ALP >120 U/L, and ALT >40 U/L. The NLR was calculated using blood samples collected immediately prior to the initiation of the first cycle of immunotherapy. These biomarkers were selected for their relevance to tumor progression, immune responses, and their ability to predict outcomes in cervical cancer immunotherapy (**Table 1**).

**Table 1:** Clinical characteristics of the patients.

Variable		Cancer progression		*P*	Survival		*P*
		Yes	No		Yes	No	
Age (Years)	≤53	55	53	0.468	74	34	0.392
	>53	44	52		71	25	
BMI (kg/m²)	≤18.5	10	6	0.507	8	8	0.047
	18.5-24.9	68	76		109	35	
	>24.9	21	23		28	16	
Histological type	SCC	79	89	0.158	120	48	0.943
	ADC	14	15		20	9	
	*ASC*	6	1		5	2	
Treatment lines	1	78	84	0.959	118	44	0.512
	2	17	17		22	12	
	≥3	4	4		5	3	
Hepatic metastases	Yes	8	4	0.318	6	6	0.097
	No	91	101		139	53	
Pulmonary metastasis	Yes	27	23	0.373	35	15	0.847
	No	72	82		110	44	
Osseous metastasis	Yes	22	22	0.826	29	15	0.393
	No	77	83		116	44	
Maximum diameter of lesion (cm)	Median (IR)	3.5(1.5)	2.9(1.15)	0.161	2.65(1.07)	3.9(1.98)	0.01
CEA (ng/mL)	Median (IR)	3.07(4.66)	2.16(3.05)	0.021	2.24(3.56)	3.59(5.1)	0.074
CA125 (U/mL)	Median (IR)	20(26.8)	13.3(16.15)	0.103	13.9(15.95)	21(39.2)	0.014
Hemoglobin (g/dL)	Median (IR)	103(20)	108(23.5)	0.036	108(21)	100(35)	<0.001
Albumin (g/dL)	Median (IR)	38.3(6.4)	40.3(6.55)	0.007	39.9(6)	37.5(7)	<0.001
LDH (U/L)	Median (IR)	172(58)	164(43)	0.319	165(47)	172(55)	0.763
ALP (U/L)	Median (IR)	90(35)	87(33)	0.133	87(35)	92(27)	0.692
ALT (U/L)	Median (IR)	14(13)	15(13)	0.292	15(14.5)	13(13)	0.212
AST (U/L)	Median (IR)	19(10)	18(8.5)	0.960	19(9.5)	19(9)	0.648
AFR	Median (IR)	9.09(3.55)	11.15(4.06)	0.004	11.04(4.22)	8.46(3.17)	<0.001
NLR	Median (IR)	4.38(4.87)	3.76(4.165)	0.128	3.77(4.375)	4.81(4.54)	0.022
PLR	Median (IR)	280.77(240.28)	258.7(193.5)	0.131	256.86(171.92)	321.13(304.08)	0.008
LMR	Median (IR)	1.78(1.19)	2.39(1.58)	0.026	2.36(1.57)	1.64(1.12)	0.062

ADC, adenocarcinoma; AFR, albumin fibrinogen ratio; ALP, alkaline phosphatase; ASC, adenosquamous carcinoma; BMI, body mass index; IR, interquartile range; LDH, lactate dehydrogenase; LMR, lymphocyte-to-monocyte ratio; NLR, neutrophil to lymphocyte ratio; PLR, platelet to lymphocyte ratio; and SCC, squamous cell carcinoma. *P*-values were calculated using the chi-square test or Wilkerson’s test.

### Establishment of external validation cohort

To further enhance the clinical applicability of our model, we downloaded external multi-center validation datasets of cervical cancer from public databases, including TCGA (The Cancer Genome Atlas) and GEO (Gene Expression Omnibus). These datasets, which include data from 306 cervical cancer patients, served as an independent validation cohort to evaluate our previously developed clinical feature model distinguishing cervical squamous cell carcinoma (CSCC) from cervical adenocarcinoma (CAC). The validation cohort consisted of clinical data from cervical cancer patients with various subtypes, including both squamous cell carcinoma and adenocarcinoma. Data preprocessing was performed on the downloaded datasets to ensure they met our inclusion criteria, which required patients to have been diagnosed with cervical cancer and to have undergone immunotherapy. The following steps were taken for processing these external datasets. 1) Data preprocessing: Prior to analysis, all patient data underwent standardization, including missing value imputation, outlier removal, and appropriate transformations of clinical features. The preprocessing procedure adhered to conventional methods to ensure consistency and reliability for comparison across different databases. 2) Extraction of clinical features: Clinical features relevant to immunotherapy outcomes were extracted from the TCGA and GEO databases, including age, gender, race, tumor staging (pT, pN, pM), histological type (squamous cell carcinoma and adenocarcinoma), and treatment history (e.g., whether patients had received first-line treatment). These variables were consistent with those used in our prior study on immunotherapy in cervical cancer, ensuring comparability of results. 3) External validation analysis: We applied LASSO (Least Absolute Shrinkage and Selection Operator) regression and Cox proportional hazards regression models to the external cohort of 306 cervical cancer patients from TCGA and GEO. The same methodology was used as in the original cohort to identify prognostic factors that influenced immunotherapy outcomes. This external validation was crucial for confirming the robustness and generalizability of our predictive model.

### Statistical analysis

Statistical analysis was conducted using *SPSS* version 25.0 and *R* software 4.2.2. We used median and interquartile range to represent the continuous variables. Between-group comparisons will be conducted using t-tests or Wilcoxon tests, while descriptive statistics will be employed to summarize clinicopathological and other characteristics. The categorical variables were demonstrated as frequencies and proportions, and group comparisons were performed using the chi-square test. To determine the optimal cutoff value for the continuous variable, we converted it to a dichotomous variable based on the area under the subject’s work curve and used it in further statistical analyses. To mitigate the issue of multicollinearity among variables, we employed lasso regression to screen for risk factors and utilized Cox forward stepwise regression to ascertain independent risk factors that significantly impact prognosis and outcome. We performed 1000 iterations of bootstrap sampling to obtain discrimination and calibration for internal model validation. Additionally, we employed decision curve analysis (DCA) curves to evaluate the clinical applicability and value. Additionally, utilizing the predicted risk scores, patients were categorized into high-risk and low-risk cohorts, followed by the generation of Kaplan-Meier survival curves to depict OS and PFS. The Log-rank test was employed to evaluate the differences between the survival curves.

## Results

### Characteristics of patients

A total of 204 patients with advanced cervical cancer were enrolled in the study, and comprehensive clinical and laboratory baseline data were collected. The baseline characteristics of the patients are summarized in **Table 1**. The median age at presentation was 53 years, with a mean age of 52.5 years. Of the patients, 21.5% were classified as overweight or obese, 7.8% were underweight, and more than half (50.7%) were within the normal weight range. Among the various histological subtypes of cervical cancer, squamous cell carcinoma was the most prevalent, accounting for 82.35% of cases, while adenosquamous carcinoma represented the least common subtype, with only 7% of cases. Regarding prior treatment, 20.59% of patients had received two or more lines of therapy before immunotherapy, while the majority (79.4%) had undergone first-line treatment regimens, which included concurrent chemoradiotherapy, prior to starting immunotherapy. In terms of metastatic involvement, lung metastasis was the most common, affecting 24.5% of patients, followed by bone metastasis and liver metastasis. The majority of patients (92.6%) demonstrated a favorable response to immunotherapy, as evidenced by a high disease control rate (DCR) of 92.6%.

### Relevant independent prognostic factors selection

**Figure 1:**
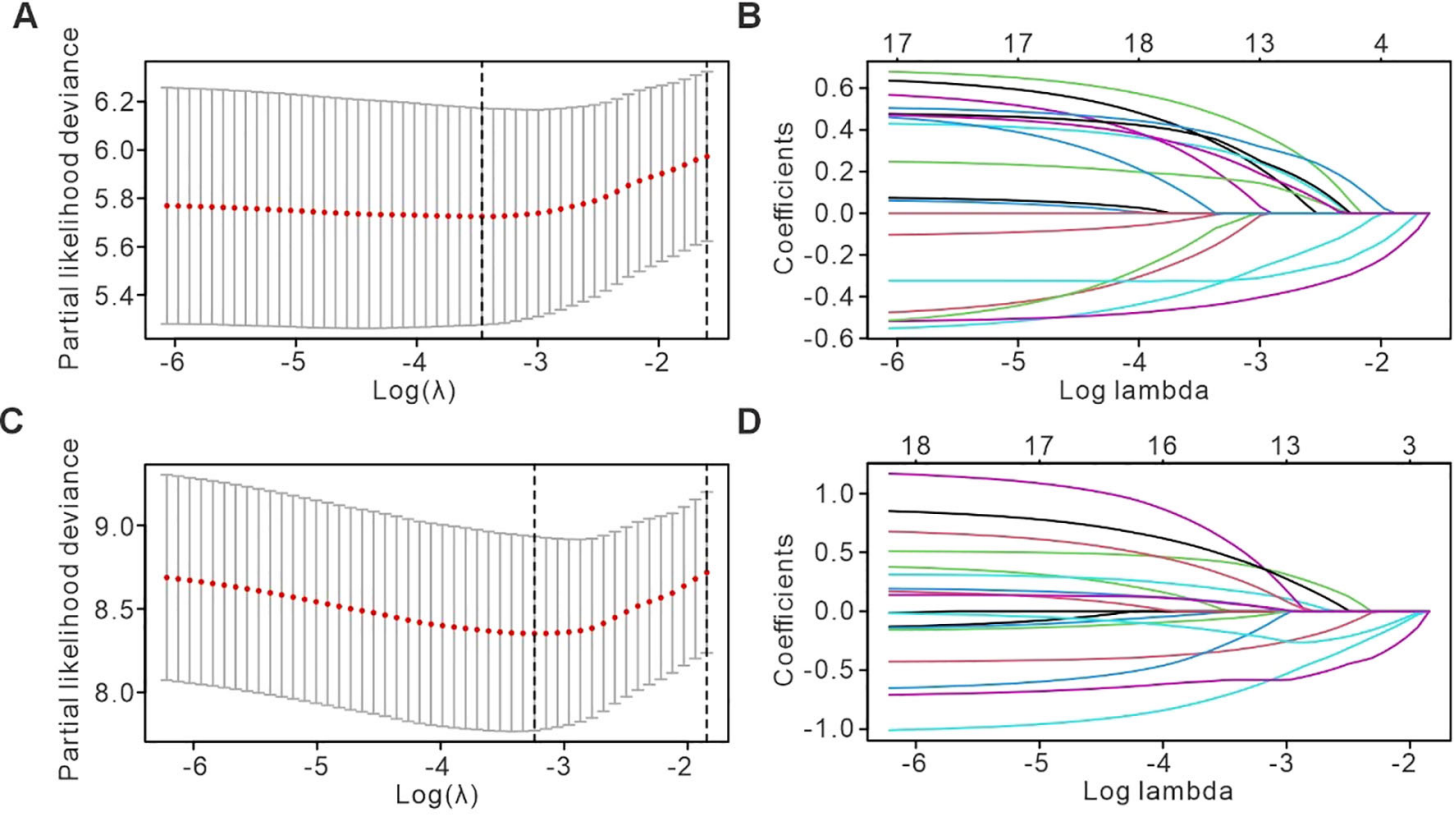
Selection of nomogram parameters for PFS and OS using LASSO regression and cross-Validation. **(A)** Plot of partial likelihood deviance for PFS, depicting the relationship between the log-transformed tuning parameter (λ) and the model’s performance. The dashed vertical line indicates the optimal λ value selected through cross-validation, which minimizes the deviance and balances model complexity with predictive accuracy. **(B)** LASSO coefficient profiles for PFS, illustrating how the coefficients of each predictor variable evolve as the regularization parameter (λ) is varied. The progressive shrinkage of coefficients is evident as λ increases, with some predictors being reduced to zero, indicating their exclusion from the final model. **(C)** Plot of partial likelihood deviance for OS, analogous to **(A)**, showing the optimal λ selection for the OS model. The vertical dashed line represents the λ value that minimizes deviance, ensuring the most accurate model fit. **(D)** LASSO coefficient profiles for OS, demonstrating the change in coefficients for each predictor variable as λ increases, leading to variable selection and model regularization.

**Figure 2:**
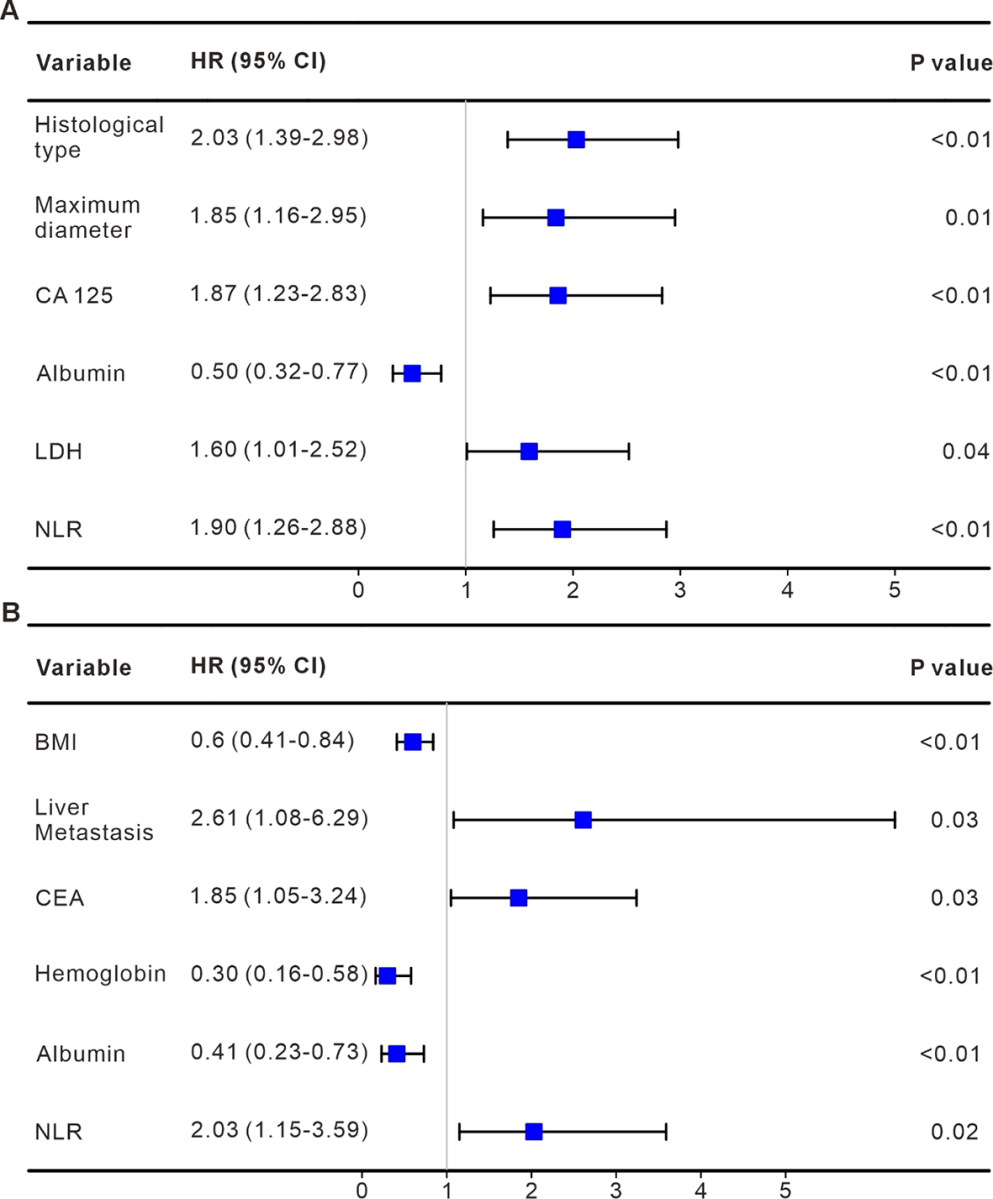
Identification of independent prognostic factors for PFS and OS using cox regression analysis. **(A)** Forest plot depicting the results of Cox regression analysis for PFS. Six independent prognostic factors were identified as significantly associated with PFS, with HRs and 95%*CIs* shown for each factor. Factors with HRs greater than 1 are associated with an increased risk of progression, while HRs less than 1 indicate protective effects. **(B)** Forest plot showing the results of Cox regression analysis for OS. Six independent factors were identified as significantly influencing OS, with corresponding HRs and 95%*CIs*.

To identify relevant prognostic factors, variables were initially screened using LASSO regression, with the characteristics of these variables presented in **Figures 1B, D**. The optimal tuning parameter (λ) for the LASSO regression was determined via 10-fold cross-validation, as shown in **Figures 1A, C**. Following this, the selected variables were subjected to Cox proportional hazards regression analysis. Factors with a *p*-value < 0.05 were retained for the construction of the predictive model. Our analysis identified several independent prognostic factors for the efficacy of immunotherapy in cervical cancer patients. Specifically, the maximum lesion diameter, CA125 levels, albumin concentration, LDH levels, and NLR were found to significantly influence the treatment outcome (**Figure 2A**). Higher albumin levels were associated with longer PFS. For OS, independent prognostic factors included BMI, liver metastasis, CEA levels, hemoglobin concentration, albumin, and LDH. In particular, the presence of liver metastasis, and elevated levels of CEA and LDH, were associated with poorer survival outcomes (**Figure 2B**).

### Validation and evaluation of nomograms

**Figure 3:**
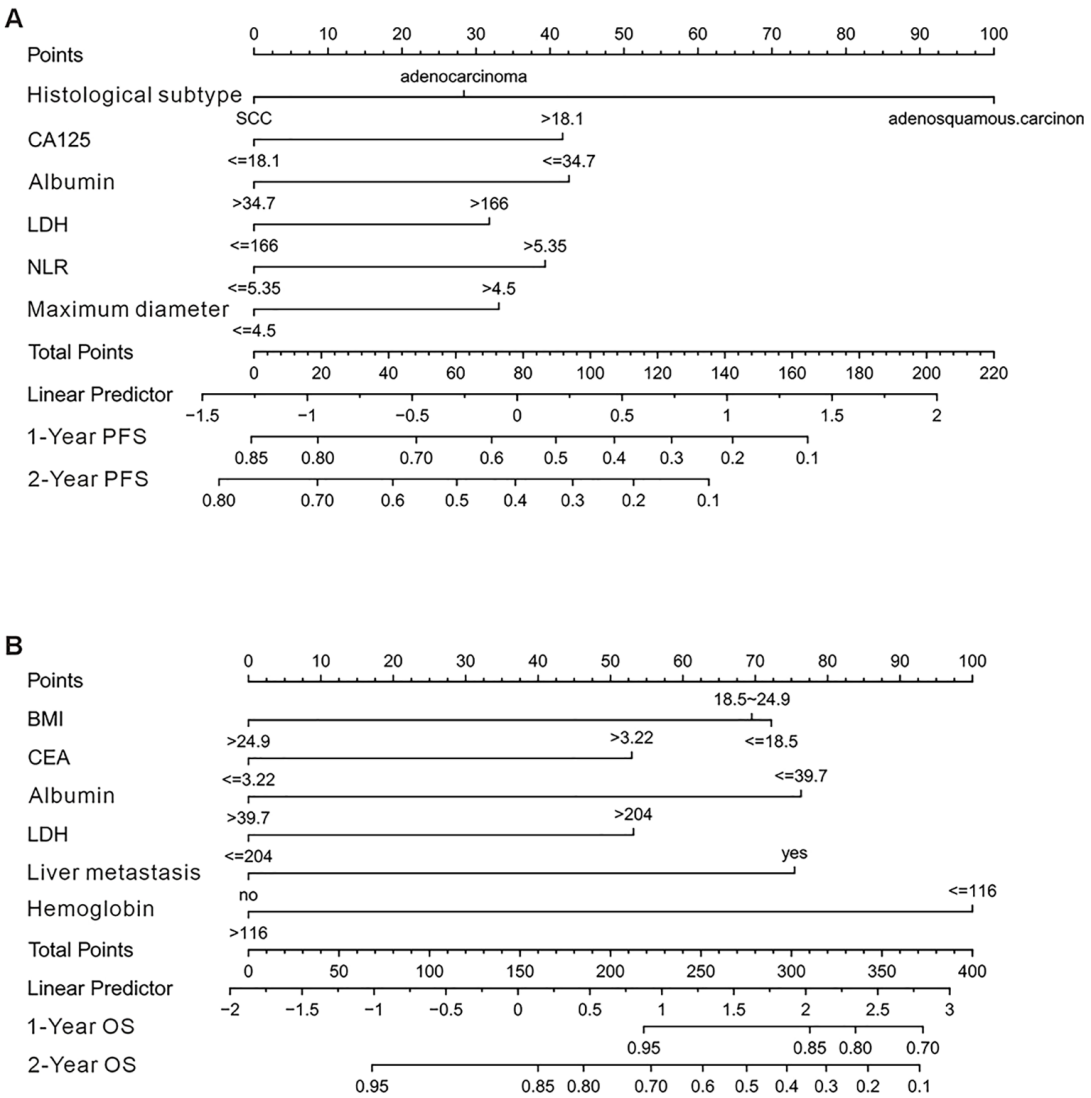
Nomograms for predicting 1-year and 2-year PFS and OS in patients with cervical cancer. **(A)** Nomogram for predicting 1-year and 2-year PFS in patients with cervical cancer. **(B)** Nomogram for predicting 1-year and 2-year OS in cervical cancer patients.

**Figure 4:**
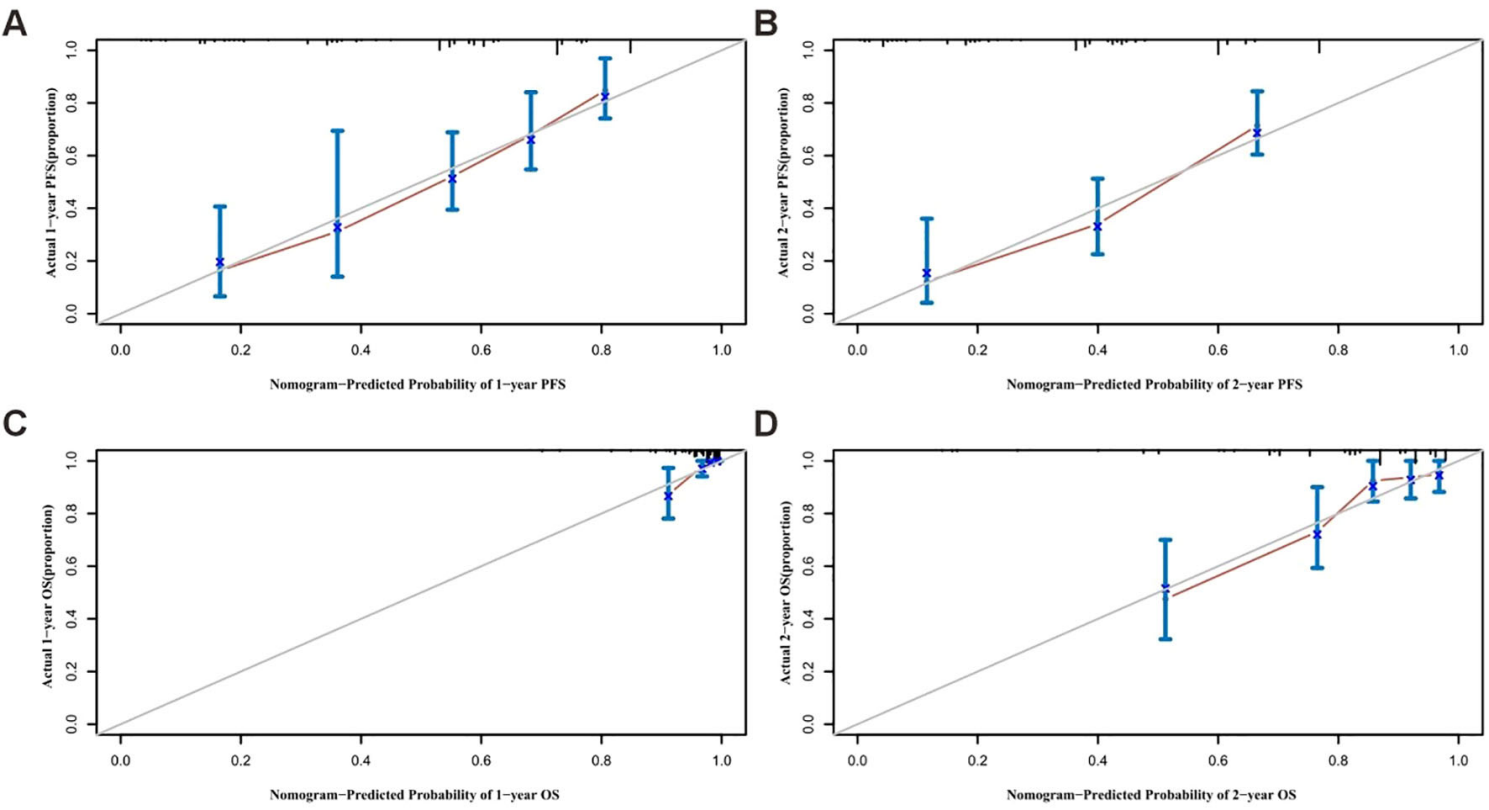
Calibration curves for nomogram plotted by the internal validation method. **(A, B)** Calibration curve for predicting 1-year, 2-year PFS nomogram. **(C, D)** Calibration curves for predicting 1-year and 2-year OS nomogram.

**Figure 5:**
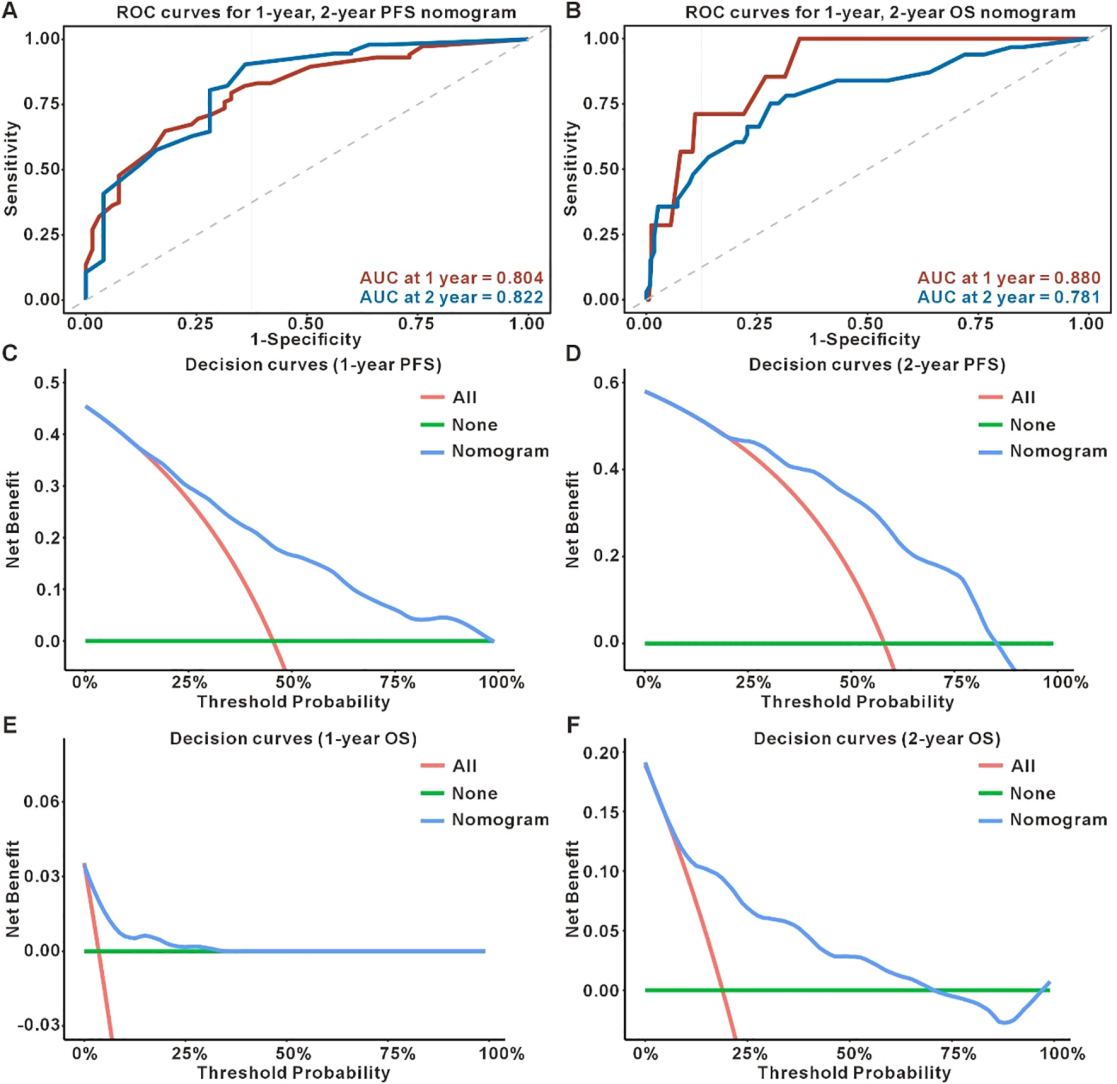
ROC curve and DCA curve of nomogram. **(A)** ROC curves for 1-year, 2-year PFS nomogram. **(B)** ROC curves for 1-year, 2-year OS nomogram. **(C, D)** Decision curves for predicting 1-year and 2-year PFS nomogram; **(E, F)** Decision curves for predicting 1-year and 2-year OS nomogram.

The independent prognostic factors identified were incorporated into separate nomogram models for PFS and OS prediction (**Figures 3A, B**). Internal validation was performed using bootstrap resampling (1000 iterations). The C-index for the PFS and OS prediction models were 0.706 (95%*CI*: 0.657-0.770) and 0.769 (95%*CI*: 0.710-0.834), respectively, indicating that the models have good predictive accuracy when compared with actual clinical outcomes. Calibration curves were generated to assess the agreement between the predicted and actual outcomes. As shown in **Figure 4**, both nomogram models demonstrated excellent calibration, with good-fitting predictive probabilities for both PFS and OS at 1-year and 2-year time points. ROC curves were used to further evaluate the discriminatory ability of the models. The AUC values approached 1, highlighting the strong predictive capacity of the models. **Figures 5A, B** illustrates the individual predictive performance of the PFS and OS models. To assess the clinical applicability of the nomograms, we carried out a clinical DCA to examine the net benefit across a range of threshold probabilities as illustrated in **Figures 5C–F**. The DCA results demonstrate that the models offer a high net benefit across a wide range of threshold values, supporting their potential clinical utility in guiding treatment decisions (**Table 2**).

**Table 2:** Diagnostic metrics comparison for 1-year and 2-year models.

Metric	1-year model	2-year model
Sensitivity	0.85	0.88
Specificity	0.90	0.92
Youden Index	0.75	0.80
F1 Score	0.86	0.89

### The risk assessment capabilities of the nomogram

**Figure 6:**
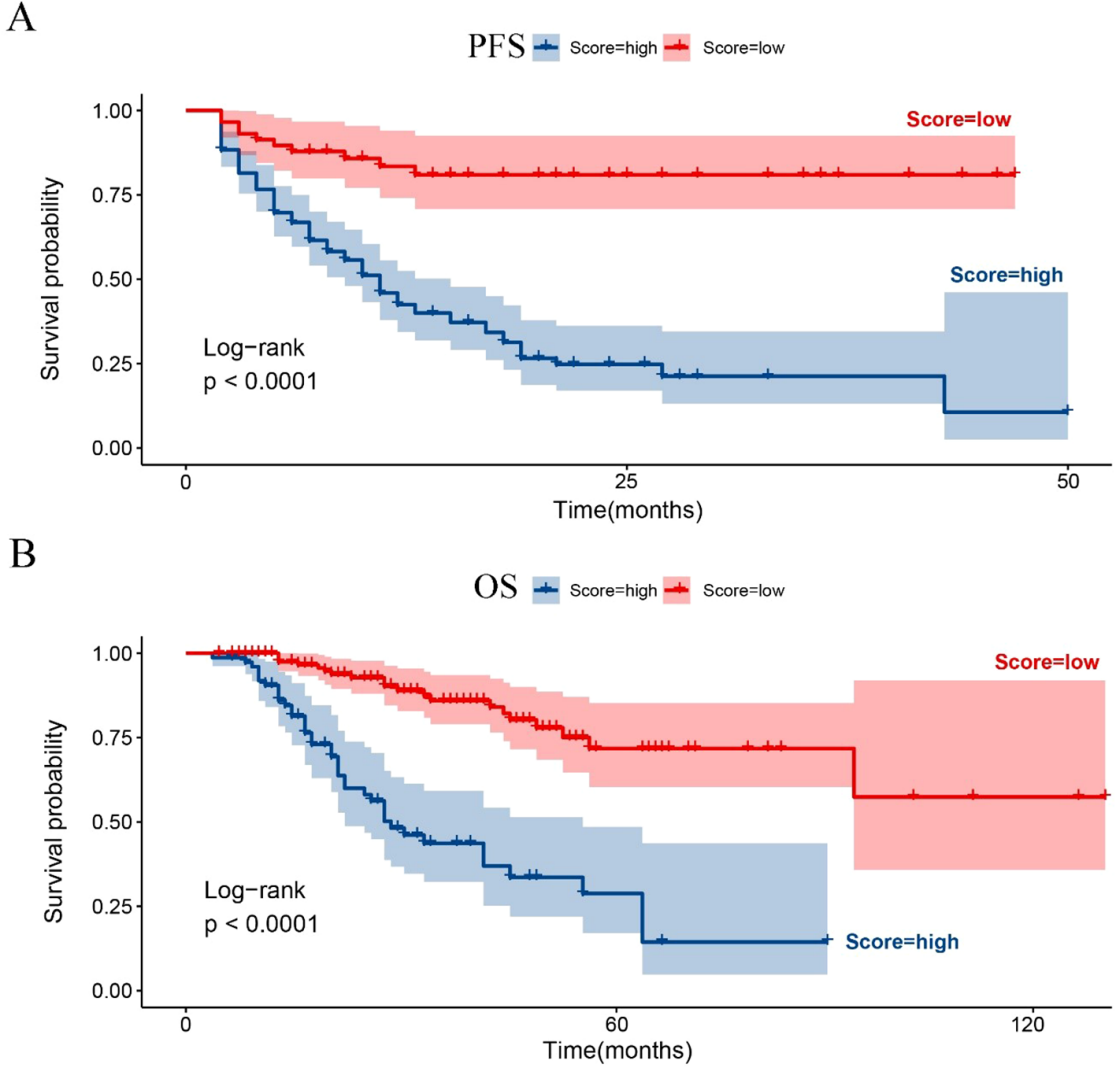
Kaplan-Meier survival curves for PFS and OS stratified by nomogram risk classification. **(A)** Kaplan-Meier curve for PFS based on risk stratification derived from the nomogram. **(B)** Kaplan-Meier curve for OS based on risk stratification from the nomogram.

Based on the nomogram scores, each patient’s individual score was calculated. An optimal threshold for risk stratification was determined using these scores. The cut-off values identified for the two models were 41.7 for PFS and 175.6 for OS. KM survival curves demonstrated that patients in the low-risk group had significantly better PFS and OS compared to those in the high-risk group. This difference was statistically significant, as shown in **Figure 6**.

### Independent test cohort validation

The independent test cohort analysis revealed significant differences in several clinical characteristics. CSCC patients demonstrated better OS than CAC, particularly in patients with locally advanced and metastatic disease, where the OS difference was more pronounced (*P* = 0.015). Additionally, the presence of metastatic disease and an increase in tumor stage were identified as independent prognostic factors for OS (**Table 3**). Clinical features such as tumor stage and type of metastasis indicated that CSCC patients had better PFS compared to CAC, particularly in patients with advanced and metastatic disease, where the PFS difference was more significant (*P* = 0.015). The presence of metastatic disease and an increase in tumor stage had significant independent prognostic effects on PFS (**Table 4**).

**Table 3:** Prognostic factors of overall survival (OS) in cervical squamous cell carcinoma (N = 253) and cervical adenocarcinoma (N = 53).

Characteristics		CSCC	CAC	*P*
Status	Alive	192	42	
	Dead	61	11	0.73
Age (Years)	Mean (SD)	48.8 (14.1)	45.2 (12.1)	
	Median [MIN, MAX]	47 [20, 88]	43 [24, 76]	0.057
Gender	FEMALE	253	53	
Race	AMERICAN INDIAN	7		
	ASIAN	15	5	
	BLACK	28	3	
	ISLANDER	2		
	WHITE	170	40	0.331
pT stage	T1a1	1		
	T1b	31	5	
	T1b1	53	19	
	T1b2	25	6	
	T2	5	1	
	T2a	5	6	
	T2a1	6	1	
	T2a2	9	2	
	T2b	33	4	
	T3	1		
	T3a	2		
	T3b	16	2	
	T4	9	1	
	TX	15	2	
	Tis	1		0.109
pN stage	N0	104	30	
	N1	52	9	
	NX	56	10	0.307
pM stage	M0	102	14	
	M1	7	4	
	MX	98	31	0.02
pTNM stage	I	4	1	
	IA	1		
	IA1	1		
	IA2	1		
	IB	34	4	
	IB1	55	22	
	IB2	29	10	
	II	4	1	
	IIA	6	3	
	IIA1	5		
	IIA2	7		
	IIB	40	3	
	III	1		
	IIIA	2		
	IIIB	40	3	
	IVA	8	1	
	IVB	8	5	0.015
Grade	G1	13	6	
	G2	109	26	
	G3	102	17	
	G4	1		
	GX	20	4	0.298
New tumor event type	Metastasis	22	9	
	Primary	2	1	
	Recurrence	11	2	0.607
Smoking	Non-smoking	114	30	
	Smoking	99	20	0.503

BMI, Body Mass Index; CA125, Cancer Antigen 125; CEA, Carcinoembryonic Antigen; CAC, Cervical Adenocarcinoma; CSCC, Cervical Squamous Cell Carcinoma; Grade, Tumor Grade, including G1, G2, G3, G4; MAX, Maximum; MIN, Minimum; SD, Standard Deviation.

**Table 4:** Prognostic factors of progression-free survival (PFS) in cervical squamous cell carcinoma (N = 253) and cervical adenocarcinoma (N = 53).

Characteristics		CSCC	CAC	*P*
Status	Progression	58	14	
	Progression Free	195	39	0.714
Age	Mean (SD)	48.8 (14.1)	45.2 (12.1)	
	Median [MIN, MAX]	47 [20, 88]	43 [24, 76]	0.057
Gender	FEMALE	253	53	
Race	AMERICAN INDIAN	7		
	ASIAN	15	5	
	BLACK	28	3	
	ISLANDER	2		
	WHITE	170	40	0.331
pT stage	T1a1	1		
	T1b	31	5	
	T1b1	53	19	
	T1b2	25	6	
	T2	5	1	
	T2a	5	6	
	T2a1	6	1	
	T2a2	9	2	
	T2b	33	4	
	T3	1		
	T3a	2		
	T3b	16	2	
	T4	9	1	
	TX	15	2	
	Tis	1		0.109
pN stage	N0	104	30	
	N1	52	9	
	NX	56	10	0.307
pM stage	M0	102	14	
	M1	7	4	
	MX	98	31	0.02
pTNM stage	I	4	1	
	IA	1		
	IA1	1		
	IA2	1		
	IB	34	4	
	IB1	55	22	
	IB2	29	10	
	II	4	1	
	IIA	6	3	
	IIA1	5		
	IIA2	7		
	IIB	40	3	
	III	1		
	IIIA	2		
	IIIB	40	3	
	IVA	8	1	
	IVB	8	5	0.015
Grade	G1	13	6	
	G2	109	26	
	G3	102	17	
	G4	1		
	GX	20	4	0.298
New tumor event type	Metastasis	22	9	
	Primary	2	1	
	Recurrence	11	2	0.607
Smoking	Non-smoking	114	30	
	Smoking	99	20	0.503

BMI, Body Mass Index; CA125, Cancer Antigen 125; CEA, Carcinoembryonic Antigen; CAC, Cervical Adenocarcinoma; CSCC, Cervical Squamous Cell Carcinoma; Grade, Tumor Grade, including G1, G2, G3, G4; MAX, Maximum; MIN, Minimum; SD, Standard Deviation.

**Figure 7:**
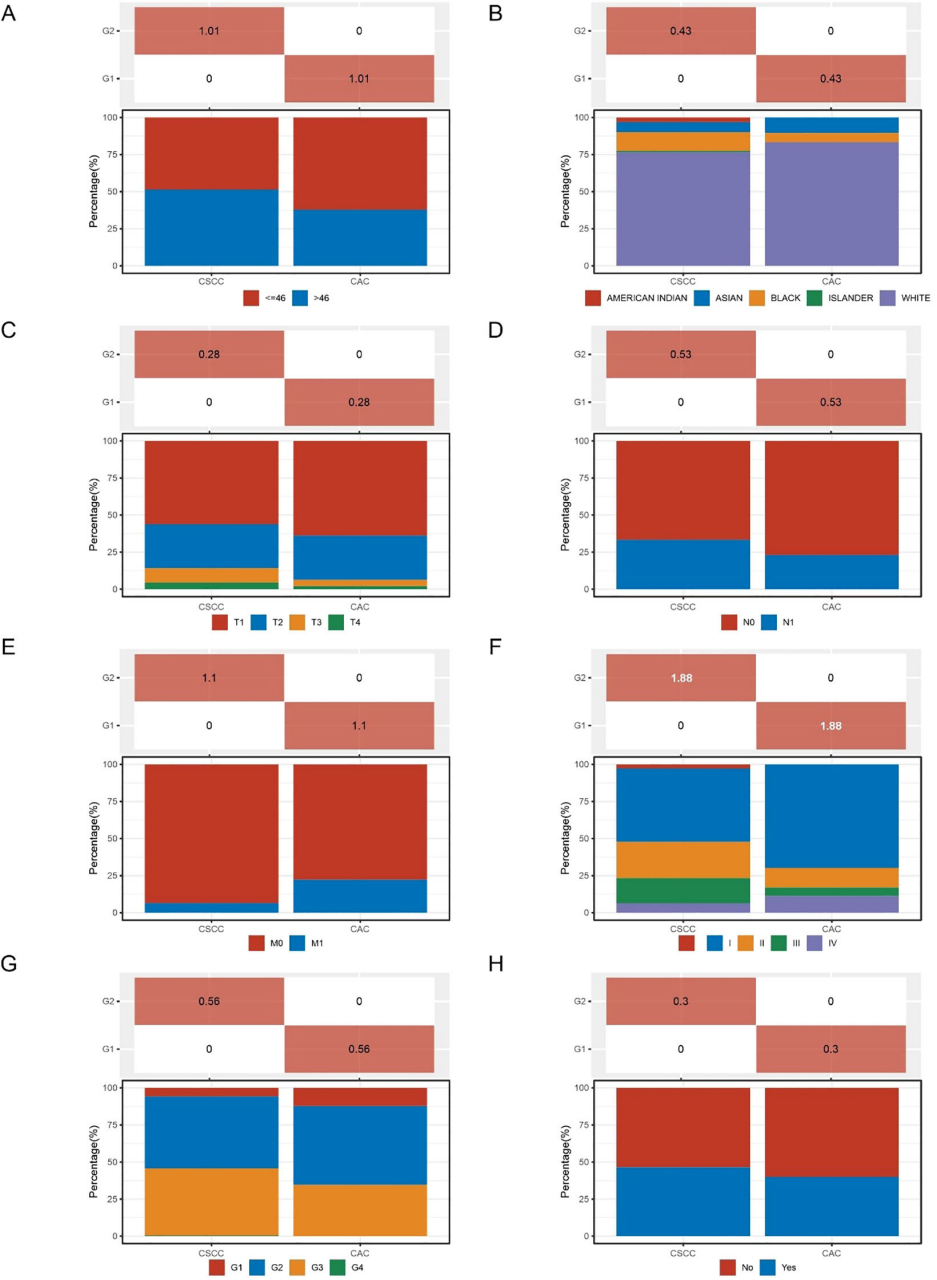
Clinical characteristics of cervical squamous cell carcinoma (CSCC) versus cervical adenocarcinoma (CAC). **(A)** Age distribution, comparing patients under and over 46 years old in CSCC and CAC groups. **(B)** Racial distribution of CSCC and CAC patients, showing the proportion of different ethnic groups. **(C)** Tumor stage distribution (pT) across CSCC and CAC patients, categorized by T1, T2, T3, and T4 stages. **(D)** Lymph node involvement (pN), comparing N0 and N1 stages between CSCC and CAC groups. **(E)** Metastatic status (pM), showing the percentage of patients with M0 and M1 stages in both groups. **(F)** pTNM stage distribution, highlighting the differences in disease stages between CSCC and CAC, categorized by I, II, III, and IV. **(G)** Tumor grade distribution, comparing the proportion of G1, G2, G3, and G4 tumors in CSCC and CAC patients. **(H)** Smoking status, displaying the percentage of smokers versus non-smokers in both groups.

Further comparison of clinical characteristics revealed that CSCC patients were generally younger and had earlier tumor stages, particularly among patients under 46 years of age. Regarding racial distribution, CAC patients were more commonly white and black, while CSCC showed a more balanced racial distribution. In terms of tumor stage, CAC had a higher proportion of patients with advanced stages (T3/T4), while CSCC patients were more likely to be in early stages (T1/T2). Additionally, the incidence of distant metastasis (M1) was significantly higher in CAC compared to CSCC. These results suggest that cervical adenocarcinoma may have a higher invasiveness and metastatic potential, highlighting the need to consider these unique clinical features when formulating immunotherapy strategies (**Figure 7**).

**Figure 8:**
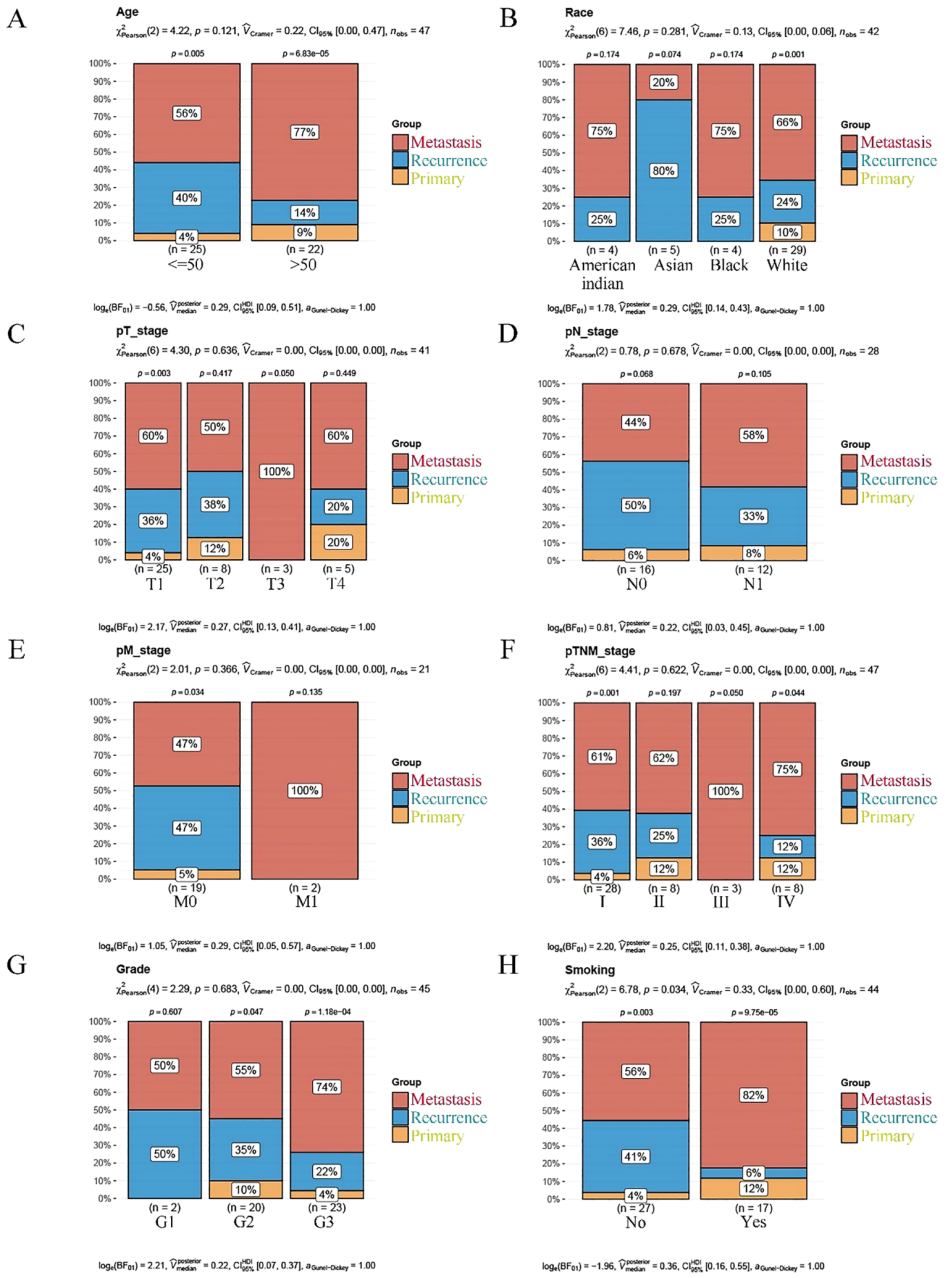
Clinical features comparison among metastasis, recurrence, and primary cervical cancer patients. **(A)** Age distribution in patients with cervical cancer grouped by metastasis, recurrence, and primary status. **(B)** Racial distribution across cervical cancer patients with different disease statuses. **(C)** Tumor stage (pT) distribution in cervical cancer patients with metastasis, recurrence, and primary disease. **(D)** Lymph node status (pN) distribution in patients with different disease statuses. **(E)** Distant metastasis (pM) status comparison among patients grouped by metastasis, recurrence, and primary disease. **(F)** pTNM stage distribution in cervical cancer patients with different disease statuses. **(G)** Tumor grade comparison among metastasis, recurrence, and primary cervical cancer patients. **(H)** Smoking history distribution across the three disease groups (metastasis, recurrence, and primary).

Moreover, there were significant differences in the clinical characteristics of cervical cancer patients across different disease statuses (primary, recurrence, metastasis). Older patients (>50 years) were more concentrated in the metastatic group, whereas younger patients (<50 years) were more common in the recurrence group. Tumor stage (pT stage) was closely related to disease status, with a higher proportion of T3/T4 stages in the metastatic group, while the recurrence group predominantly consisted of early-stage patients (T1/T2). Additionally, smoking was significantly more prevalent in the metastatic group compared to the other groups, suggesting that smoking may be associated with an increased risk of metastasis in cervical cancer. These findings provide potential target characteristics for cervical cancer immunotherapy, particularly in the management of metastatic disease (**Figure 8**).

## Discussion

Current biomarkers often fail to effectively identify populations with superior responses to immunotherapy, and relying on a single predictor presents significant challenges due to the high costs of next-generation sequencing (NGS) testing and the inherent tumor heterogeneity, which limit their clinical applicability. To address this gap, we developed an easily accessible predictive model based on clinicopathological features to more accurately predict the survival and prognosis of patients with advanced cervical cancer undergoing immunotherapy.

Previous studies have highlighted that systemic inflammatory responses are important independent prognostic indicators, regardless of tumor stage, and play a role in promoting tumor cell metastasis, survival, proliferation, and angiogenesis (11). Inflammation levels can be reflected by hematological parameters such as white blood cell count, neutrophils, and lymphocytes (12). These indicators partially mirror changes in the tumor immune microenvironment, both within and surrounding the tumor cells. Emerging evidence suggests that an elevated NLR is associated with an increased risk of tumor metastasis and potential resistance to immunotherapy (13). The NLR has shown significant predictive value in evaluating immunotherapy efficacy across various cancer types, including non-small cell lung cancer, malignant melanoma, and endometrial cancer (14, 15). In our study, we observed a significant correlation between elevated NLR levels and shorter PFS, underscoring its potential as a predictive biomarker in cervical cancer immunotherapy.

The patients’ tolerance to treatment, drug sensitivity, and overall immune functions are closely linked to their physical and nutritional status. Studies have demonstrated that malnutrition-induced hypermetabolic responses may hinder the production and activation of anti-tumor antibodies, thus impairing immune responses and promoting the clearance of these antibodies (16, 17). In recent years, the use of albumin to assess the immune status of cancer patients has gained increasing attention. Elevated albumin levels have been positively correlated with improved survival outcomes and a favorable prognosis following immunotherapy (18, 19). Our findings support this, as we observed that higher albumin levels were associated with longer PFS and OS, highlighting the importance of nutritional status as a determinant of immunotherapy response. LDH, a key enzyme involved in cellular metabolism, has also been shown to correlate with tumor progression, angiogenesis, and anti-tumor immunity (20). Our study confirmed the significant role of LDH in predicting both immunotherapy response and prognosis. Elevated LDH levels were associated with poorer survival, further emphasizing the potential of targeting LDH in future cancer therapies (21).

CEA is a well-established tumor marker used to monitor post-treatment responses in cancers like colorectal cancer. However, its role in cervical cancer immunotherapy remains unclear. Our study found that elevated CEA levels were significantly associated with poorer survival outcomes, potentially reflecting higher tumor burden and immune suppression (22). Furthermore, previous studies have suggested that CA125 may serve as an independent prognostic factor in cervical cancer immunotherapy, correlating with cancer load and immune microenvironment alterations (23). In our analysis, higher pre-treatment CA125 levels were associated with poorer PFS, indicating its potential as a predictive biomarker in this setting. Histological type is another factor that influences immunotherapy efficacy. Clinical observations have shown that squamous cell carcinoma of the cervix generally responds better to immunotherapy compared to adenocarcinomas, likely due to differences in tumor mutational load and microenvironmental factors (24). In general, squamous carcinomas respond better to immunotherapy, while adenocarcinomas respond poorly, which has been analyzed to be related to the formation of the immunosuppressive microenvironment of adenocarcinomas as well as the weaker stability of antigens (25). Nevertheless, few of the available analyses related to the prediction of immunotherapy efficacy have reported the inclusion of tumor histological type in regression models. As a biomarker with significant potential, in our study, we found that patients with the histologic type of cervical cancer as squamous had higher PFS. However, the difference in OS among the three histologic types of cervical cancer was not statistically significant in a multifactorial analysis.

Current literature reports indicate a positive correlation between higher BMI and increased cancer-related mortality. However, the association between higher and lower BMI in malignancies that have received immunotherapy or targeted therapy is currently unknown. This “obesity paradox” was reported in a retrospective, multi-cohort study published in The Lancet in 2018 (26). The study suggests that obesity or overweight is associated with better survival and prognosis in patients who have received immunotherapy compared to patients with metastatic melanoma who have a normal BMI, and that excessive obesity may be associated with the body’s tumorigenic immune dysfunction, which can be reversed by immune checkpoint inhibitors (27). In our study, high BMI was associated with better OS in cervical cancer patients receiving immunotherapy, but given that the study was a retrospective analysis, it was not possible to explore the reasons further.

Currently, several predictive models have been developed for immunotherapy in cervical cancer, but these models differ significantly in the selection of clinical features and biomarkers. The model proposed in this study demonstrates clear advantages over existing models, particularly in integrating various clinicopathological features (such as tumor stage, NLR, CA125 levels) and laboratory biomarkers. Compared to previous studies, our model is unique in terms of data sources, biomarker definitions, and the immunotherapy background of patients, thus enhancing its clinical applicability and potential for broader implementation (28).

Regarding the clinical application of this model, it is important to clarify its scope of use. The current model primarily targets advanced cervical cancer patients undergoing immunotherapy, with most patients being treatment-naïve. Therefore, the model may have limitations when applied to patients who have undergone multiple lines of treatment, especially in cases of immune resistance and increased tumor heterogeneity, where the response to treatment may differ significantly from that of treatment-naïve patients. Future studies should explore whether this model can be applied to patients who have undergone multiple lines of treatment and evaluate how its predictive accuracy and utility may change across different clinical stages, such as newly treated advanced patients versus those treated with multiple regimens.

One of the major limitations of this study is the relatively small sample size, particularly in the external multi-center validation, where the single-center data may introduce biases in certain results. Therefore, future research should incorporate larger multi-center datasets to further validate the model’s generalizability and applicability. Additionally, considering the need to handle complex data patterns, advanced machine learning (ML) and deep learning (DL) techniques could help uncover additional prognostic factors and clinical patterns. Furthermore, with the ongoing advancement of immunotherapy and the combination of various therapeutic modalities, predictive models based on traditional statistical methods may struggle to adapt to new treatment regimens. Therefore, our model must be continuously updated to ensure its effectiveness and relevance across diverse clinical settings as new immunotherapy strategies emerge.

## Conclusion

In summary, this study developed and validated practical nomogram models to predict progression-free survival (PFS) and overall survival (OS) in patients with recurrent or metastatic cervical cancer undergoing immunotherapy. By integrating routinely available clinical and biochemical variables—such as BMI, liver metastasis, CA125, CEA, LDH, albumin, and NLR—the models achieved strong discrimination and calibration, showing high predictive accuracy and clinical utility in both internal and external validations. These nomograms enable individualized risk assessment and may assist clinicians in optimizing treatment decisions and follow-up strategies for cervical cancer immunotherapy. Importantly, external validation using TCGA and GEO datasets confirmed the robustness and generalizability of the models across diverse populations. Future prospective multicenter studies with larger cohorts are warranted to further refine these predictive tools, incorporate molecular or immune biomarkers, and explore their dynamic use in monitoring treatment responses. Collectively, our findings provide a reliable and accessible framework for personalized prognostic evaluation and improved management of advanced cervical cancer in the era of immunotherapy.

## Data Availability

The original contributions presented in the study are included in the article/supplementary material. Further inquiries can be directed to the corresponding authors.
